# Electrochemical Immunosensors Based on Screen-Printed Gold and Glassy Carbon Electrodes: Comparison of Performance for Respiratory Syncytial Virus Detection

**DOI:** 10.3390/bios10110175

**Published:** 2020-11-13

**Authors:** Wioleta Białobrzeska, Daniel Firganek, Maciej Czerkies, Tomasz Lipniacki, Marta Skwarecka, Karolina Dziąbowska, Zofia Cebula, Natalia Malinowska, Daniel Bigus, Ewelina Bięga, Krzysztof Pyrć, Katarzyna Pala, Sabina Żołędowska, Dawid Nidzworski

**Affiliations:** 1 Institute of Biotechnology and Molecular Medicine, 3 Trzy Lipy St., 80-172 Gdansk, Poland; d.firganek@ibmm.pl (D.F.); m.skwarecka@ibmm.org.pl (M.S.); karolina.dziabowska@etongroup.eu (K.D.); z.cebula@ibmm.pl (Z.C.); d.bigus@ibmm.pl (D.B.); e.biega@ibmm.pl (E.B.); sabina.zoledowska@etongroup.eu (S.Ż.); dawid@etongroup.eu (D.N.); 2 SensDx, 14b Postępu St., 02-676 Warszawa, Poland; natalia.malinowska@etongroup.eu (N.M.); katarzyna.pala@etongroup.eu (K.P.); 3 Institute of Fundamental Technological Research Polish Academy of Sciences, Pawińskiego 5B, 02-106 Warszawa, Poland; mczerkie@ippt.pan.pl (M.C.); tlipnia@ippt.pan.pl (T.L.); 4 Malopolska Centre of Biotechnology, Jagiellonian University, Gronostajowa 7, 30-387 Krakow, Poland; K.a.pyrc@uj.edu.pl

**Keywords:** respiratory syncytial virus, cyclic voltammetry, electrochemical impedance spectroscopy, sensor, gold electrode, glassy carbon

## Abstract

This paper presents the development and comparison of label-free electrochemical immunosensors based on screen-printed gold and glassy carbon (GC) disc electrodes for efficient and rapid detection of respiratory syncytial virus (RSV). Briefly, the antibody specific to the F protein of RSV was successfully immobilized on modified electrodes. Antibody coupling on the Au surface was conducted via 4-aminothiophenol (4-ATP) and glutaraldehyde (GA). The GC surface was modified with poly-L-lysine (PLL) for direct anti-RSV conjugation after EDC/NHS (1-Ethyl-3-(3-dimethylaminopropyl)carbodiimide/N-Hydroxysuccinimide) activation. Electrochemical characterizations of the immunosensors were carried out by cyclic voltammetry (CV) and electrochemical impedance spectroscopy (EIS). GC-based immunosensors show a dynamic range of antigen detection from 1.0 × 10^5^ PFU/mL to 1.5×10^7^ PFU/mL, more than 1.0 × 10^5^ PFU/mL to 1.0 × 10^7^ PFU/mL for the Au-based sensor. However, the GC platform is less sensitive and shows a higher detection limit (LOD) for RSV. The limit of detection of the Au immunosensor is 1.1 × 10^3^ PFU/mL, three orders of magnitude lower than 2.85 × 10^6^ PFU/mL for GC. Thus, the Au-based immunosensor has better analytical performance for virus detection than a carbon-based platform due to high sensitivity and very low RSV detection, obtained with good reproducibility.

## 1. Introduction

The respiratory syncytial virus (RSV) belongs to the Pneumoviridae family, which includes enveloped, negative-sense single-stranded RNA viruses. The virus is decorated with the three surface glycoproteins, of which the F protein (fusion protein) is responsible for the cell entry and syncytia formation. RSV was discovered more than 50 years ago [1,2] and it has since been identified as the most common cause of acute respiratory tract infections in infants [3,4]. RSV is one of the most common respiratory pathogens in children and the elderly, with high hospitalization and mortality rates [5,6,7]. Infections are most frequent and severe in young children, causing bronchiolitis, pneumonia, or croup. In older children, adolescents, and adults, RSV infection is less severe [8]. In all age groups, RSV is a frequent cause of nosocomial diseases and may significantly impact hospitalization and healthcare costs [9]. No vaccine is available, and treatment options are limited [10].

At present, the diagnostics of acute viral infections are based mostly on RT-qPCR, and this is typically performed on respiratory samples. Some antigen detection lateral flow assays are available; however, their reliability is questioned [11,12,13,14,15,16,17,18]. While classical RT-qPCR is laborious and time ineffective, some point-of-care (POC) solutions have been developed, e.g., ID NOW RSV or Cobas Influenza A/B and RSV assay [19].

Electrochemical methods are believed as a good alternative for the gold standard methods mentioned above. They offer fast and cheap analyses with miniaturization and automation possibilities [20]. From all electrochemical techniques, electrochemical impedance spectroscopy (EIS) is the most beneficial thanks to real-time reaction monitoring. Additionally, it has a non-destructive impact on analyzed samples, low current demand, and high sensitivities of sensor performance [21,22]. EIS is commonly used for the detection of other respiratory viruses, like influenza [23,24,25].

Electrochemical immunosensor development employs antibodies as recognition elements. The sensitivity of the assay often relies on this protein and its attachment to a solid platform [26]. The proper choice of linkers for Ab immobilization is crucial for further sensitivity in antigen recognition. For gold electrodes, the sulfur-ended linker is preferable due to self-assembled monolayer formation [26]. Exemplary are 4-aminothiophenol (4-ATP) or cysteamine, which have a free amine group that can react with the carboxylic group of antibodies. Carbon-based electrodes, e.g., glassy carbon (GC) or boron-doped diamond (BDD), are electrochemically modified to form chemically reactive groups [27]. Exemplary are the reduction of diazonium salt [28] or poly-L-lysine [29], which form covalent bonds with the carbon surface.

**Figure 1:**
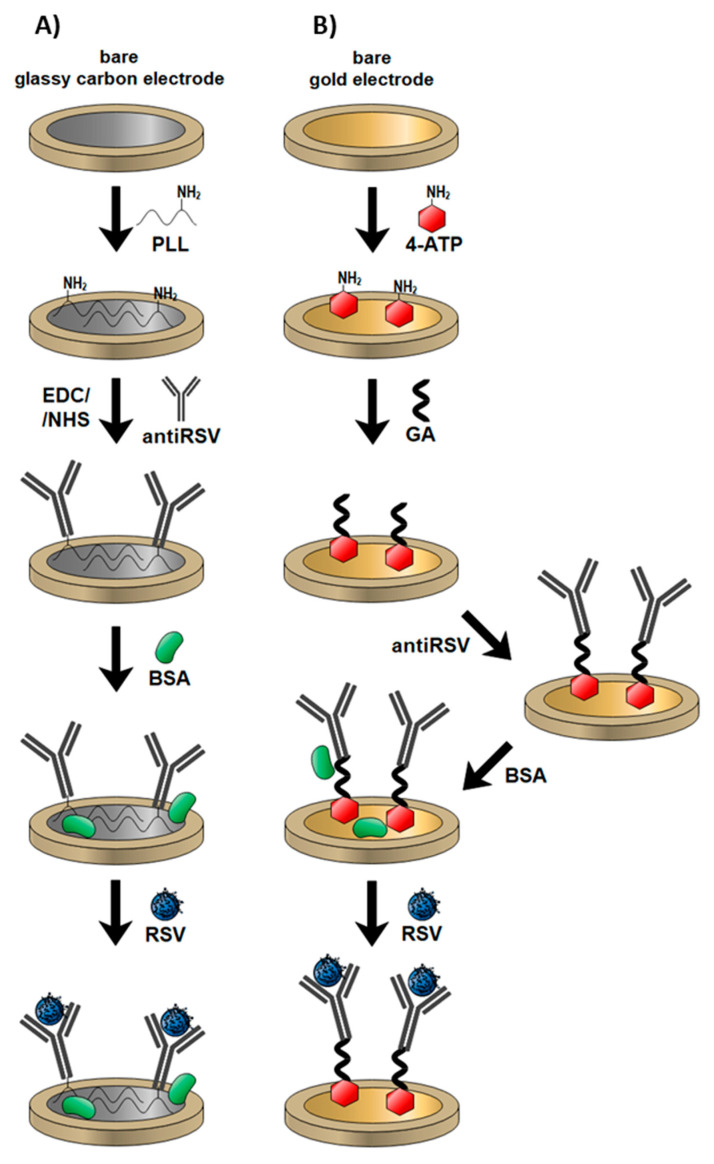
Mechanisms of different electrode modifications with anti-respiratory syncytial virus (RSV) antibodies and RSV detection: (**A**) gold electrode; (**B**) glassy carbon (GC) electrode.

In this work, we first report the development and comparison of two label-free impedimetric immunosensors for the rapid detection of RSV. The assay is based on two commercially available electrode materials manufactured with various technologies, screen-printed disposable gold and glassy carbon reusable discs. They were modified with anti-RSV using different approaches and the antibody specific to the F protein of RSV was successfully immobilized on the Au surface via 4-ATP and glutaraldehyde (GA) linkers, while the GC surface was modified with poly-L-lysine for direct anti-RSV conjugation after N-Ethyl-3-(3-dimethylaminopropyl)carbodiimide/N-Hydroxysuccinimide (EDC/NHS) activation of its carboxylic groups (Figure 1). The performance of produced biosensors was checked by EIS. The detection procedure is fast (5 min detection) and no sample processing is required. Both systems were compared in terms of detection limits (LODs), linear ranges of antigen detection, and selectivity.

## 2. Materials and Methods

For electrode preparation and modification, 99.8% ethanol and sulfuric acid were provided by Chempur (Piekary Slaskie, Poland); phosphate-buffered saline (PBS) tablets, 97% 4-aminothiophenol (4-ATP), 25% glutaraldehyde (GA), N-ethyl-N′-(3-dimethylaminopropyl)carbodiimide hydrochloride (EDC), 98% N -hydroxysuccinimide (NHS), 0.1% poly-L-lysine (PLL), and bovine serum albumin (BSA) were provided by Sigma-Aldrich (Munich, Germany). HeLa cells were cultured on Dulbecco’s modified Eagle’s medium (4.5 g/L of D-glucose and 0.1 mM L-glutamine; Thermo Fisher, Warsaw, Poland) supplemented with 10% fetal bovine serum (FBS) and a 200 µg/mL penicillin/streptomycin mix. Cells were cultured at 37 °C in an atmosphere containing 5% CO_2_ and were passaged upon reaching 90% confluency (every 2–3 days). PEG6000 (BioUltra grade) for precipitation of viral particles was purchased from Sigma-Aldrich. The RNA was extracted by ExtractMe Viral RNA and DNA kit (Blirt, Gdansk, Poland), and concentration was measured by a NanoDrop One (Thermo Scientific, Warsaw, Poland). The RT-qPCR reaction was carried out by a SensiFAST Probe No-ROX One-Step kit (Bioline), with 15 pmol of each of the primers (RS-1 AACAGATGTAAGCAGCTCCGTTATC; RS-2CGATTTTTATTGGATGCTGTACATTT) and 5 pmol of the probe (RS-3 FAM-TGCCATAGCATGACACAATGGCTCCT -BHQ-1) (Sigma-Aldrich, Poznan, Poland). The reaction was carried out in a CFX Connected thermocycler (Bio-Rad, Warszawa, Poland) with the profile: 1 cycle for 10 min at 48 °C and 3 min at 95 °C, followed by 45 cycles for 15 s at 95 °C and 40 s at 60 °C.

### 2.1. Electrochemical Procedures

The cyclic voltammetry (CV) and EIS measurements were conducted using a Palmsens4 potentiostat/galvanostat system (Methrom, Autolab, the Netherlands) in the standard three-electrode configuration. A screen-printed gold electrode (4 mm diameter, DropSens, Spain) or glassy carbon disk electrode (3 mm diameter, Mineral, Poland) was used as a working electrode and Pt mesh served as a counter electrode, while Ag/AgCl/0.1 M KCl was used as a reference electrode. All the electrochemical tests were carried out in 5 mM K_3_[Fe(CN)_6_]/0.01 M PBS aqueous solution (pH 7.45), that was previously deaerated. In the EIS measurements, the frequency ranged from 10 kHz to 1 Hz with 41 points. The amplitude of the AC signal was 10 mV. Obtained impedance spectra were recorded at the redox reaction formal potential (EF). The EF value was calculated based on the redox peak positions on the CV voltammograms for the gold electrode and glassy carbon electrode independently. Each potential was held constant for 60 s before each measurement to obtain steady-state conditions. Obtained data were subjected to analysis using EIS Spectrum Analyzer according to the proposed electric equivalent circuit (EEQC).

### 2.2. Preparation of Au/4-ATP/Anti-RSV/BSA Immunosensor

The cleaning of the bare gold surface is critical for self-assembled monolayer formation and should be accomplished systematically. The gold surface was first polished with a 0.05 µm alumina slurry, and the electrode was washed with a large amount of deionized water. Pretreated gold electrodes were immersed in 0.1 M 4-ATP in ethanol for 12 h to form a self-assembled monolayer (SAM). The substrates were rinsed with ethanol to remove the unbonded thiols. To convert the terminal amine groups, the thiol-modified electrodes were incubated in 2.5% glutaraldehyde for 15 min and protected from light. Afterward, gold electrodes were rinsed with water and dried under an argon stream. The electrode’s surface was overlaid with 10 µg/mL of monoclonal anti-RSV IgG and incubated at 37 °C for 1 h. The fluid was removed, and the electrodes were rinsed with PBS. Then, electrodes were treated with 0.1% BSA for 30 min to block the non-specific binding sites. After rinsing with the PBS and water, electrodes were dried under an argon stream. The mechanism of the electrode coating is summarized in Figure 1.

### 2.3. Preparation of GC/PLL/Anti-RSV/BSA Immunosensor

The bare GC electrodes were cleaned mechanically with 1 µm and 0.05 µm alumina slurry and rinsed profusely with deionized water. Poly-L-lysine was then electropolymerized on each GC electrode surface using a CV technique with a potential sweep between 0.5 V and 1.5 V versus Ag/AgCl at 100 mV/s for 15 cycles. Electropolymerization was carried out in a solution containing 117 µL PLL in 7 mL PBS (pH 7.45). Subsequently, a 10 μg/mL solution of monoclonal anti-RSV IgG in PBS containing EDC and NHS was dropped on the dry GC electrode surface and left at 4 °C for 90 min. A 40 mM EDC/NHS mixture was used to activate IgG antibodies’ terminal carboxylic groups for direct coupling with amine groups present on poly-L-lysine-coated electrodes. Residual unmodified sites were blocked by incubation in 0.1% BSA solution at 4 °C for 30 min. Finally, the electrodes were rinsed with water and dried with an argon stream and were ready for further use. The schematic representation of GC-based immunosensor fabrication is shown in Figure 1.

### 2.4. Virus Propagation

**Figure 2:**
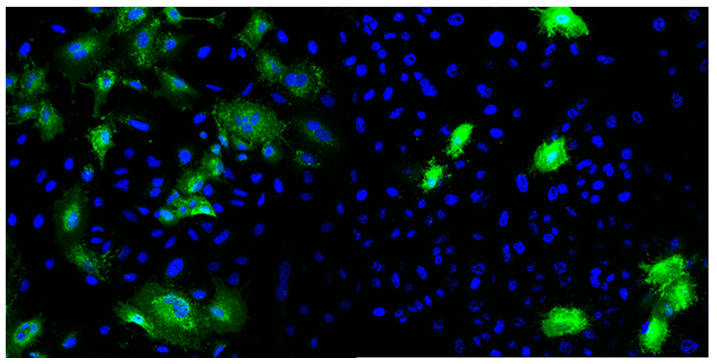
Example of HeLa cells with stained nuclei (blue) and RSV F protein (green) after 24 h of infection.

The respiratory syncytial virus (RSV) A2 strain was purchased from ATCC and amplified in HeLa cells. Cells were seeded in 225 cm^2^ tissue culture flasks (Falcon, Lodz, Poland) and cultured for 2–3 days until reaching 90% confluency. Example of HeLa cells with stained nuclei (blue) and RSV F protein (green) after 24 h of infection are represented in Figure 2. The medium was removed, cells were washed once with PBS, and overlaid with 15 mL of PBS-diluted RSV at an MOI of 0.1. The infection was carried out for 2 h at 37 °C. Then, DMEM medium supplemented with 2% FBS was added to a total volume of 40 mL per flask. Infected cells were cultured for 3–5 days until the cytopathic effect was evident for ~80% of the cells. Virus-containing culture supernatant was collected and clarified by centrifugation at 3000× *g*, at 4 °C, for 20 min. Virus particles were concentrated by precipitation with 50% (W/V) PEG6000 (Sigma-Aldrich) in NT buffer (150 mM NaCl, 50 mM Tris-HCl, pH 7.5) and stirred gently at 4 °C for 90 min. The virus was centrifuged at 3250× *g* at 4 °C for 20 min and re-centrifuged after removing supernatant to remove the remaining fluid. The pellet was suspended in 1 mL of NT buffer with 100 mM MgSO_4_, aliquoted, and stored at −80 °C. Each aliquot was used only once due to significant loss of virus activity during repeated freeze–thaw cycles.

No further purification of the virus was performed to avoid the addition of sucrose and/or iodixanol into the viral stock, which could negatively impact the assay procedure.

### 2.5. Quantification of RSV Infectious Particles

Virus particles in collected samples were quantified using immunofluorescence. HeLa cells were seeded on microscopic coverslips and cultured upon reaching 90–100% confluency. Serial dilutions of virus samples were made in DMEM with 2% FBS in a 10^−3^ to 10^−6^ range. After washing with PBS, cells were overlaid in duplicate with the diluted virus, which was allowed to adhere for 4 h. Afterward, the virus-containing medium was removed, cells were overlaid with fresh DMEM supplemented with 2% FBS, and cultured for 24 h. Cells were washed once with PBS and fixed with 4% formaldehyde for 20 min at RT. Cells were stained using a standard immunofluorescence protocol, first with anti-RSV protein F mouse antibody (Abcam 43812) and then with an anti-mouse secondary antibody with Alexa Fluor 488 (Thermo Fisher A28175). Cells containing RSV proteins were counted using the Leica SP5 confocal microscope. Virus concentration was calculated using the following formula:

(1) $$\frac{avg.\;number\;of\;infected\;cells}{dilution\;factor\times \;volume\;containing\;virus\;added}\;=infectious\;particles\;\left[PFU/ml\right]$$

### 2.6. Virus RNA Quantification

Virus identification and quantification were performed using a one-step RT-qPCR method, according to Mentel et al. [17] using a SensiFAST Probe No-ROX One-Step Kit. Serial three-fold dilutions were prepared, and a standard curve of Ct versus PFU (plaque-forming unit) was determined (Figure S1). To quantify the PFU, 2 µL of the sample were added to reaction mixtures: 5 µL SensiFast Probe No-Rox One-Step mix, 0.4 µL of each primer (RS-1, RS-2), and 0.05 µL probe (RS-3), amplified to determine the Ct value and PFU/mL was estimated based on a standard curve.

## 3. Results

### 3.1. Characterization of the Test Electrodes

The characterization of the Au and GC electrodes’ subsequent modification steps was carried out by CV and EIS. All electrochemical measurements were performed in PBS solution, pH 7.45, containing 5 mM K_3_[Fe(CN)_6_] and 5 mM K_4_[Fe(CN)_6_]. Ferri/ferrocyanide electron transfer kinetics change at different electrode surfaces, and therefore, this redox couple was used to investigate the signal changes after each surface modification step. CV measurements were performed in the potential ranges from −0.65 V to 0.85 V (Au) and −0.3 V to 0.8 V (GC) with a scan rate of 0.05 Vs^−1^. The potential differences derive from electrochemical windows characteristic for each electrode. CV spectra inform about the charge transfer changes, and EIS records indicate the resistance changes occurring on the electrode surface.

**Figure 3:**
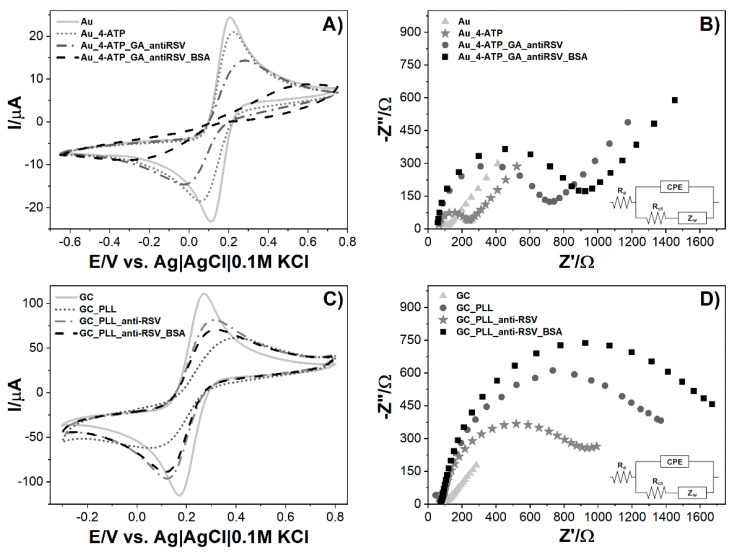
Measurements of biosensor fabrication steps: (**A**) cyclic voltammograms for the bare and modified Au electrode, (**B**) electrochemical impedance spectra for the bare and modified Au electrode, (**C**) cyclic voltammograms for the bare and modified GC electrode, (**D**) electrochemical impedance spectra for bare and modified GC electrode. Registered in 5 mM K_3_[Fe(CN)_6_]/K_4_[Fe(CN)_6_]/0.01 M PBS. Insets represent electric equivalent circuit (EEQC) utilized for fitting and data analysis.

In Figure 3, the comparison of two biosensors is presented in the case of CV (Figure 3A,C) and EIS (Figure 3B,D) measurements for every modification step. CV analysis demonstrates that bare Au and GC electrodes have reversible behavior towards the Fe^2+^/Fe^3+^ redox probe with peak-to-peak separation of 100 mV and 106.2 mV, respectively. The Au electrode’s subsequent modification tends to redox currents and decrease and increase the redox potential difference (ΔE = E_OX_ − E_RED_). Most visible changes occur after antibody binding, where the redox currents decrease significantly. This is due to the large size of the protein and its effective anchor to the previously 4-ATP-modified gold platform. The last step of free-site blockage using the small neutral protein BSA shows a drastic decrease and no characteristic current peaks, suggesting dense-packed biolayer formation. Blocking unmodified sites on the electrode surface, e.g., using BSA, is one of the most critical steps during the immunosensor fabrication [30,31,32]. It allows for the blocking of non-specific interactions. Voids in the deficient antibody overlay, covered with BSA, result in an R_ct_ increase.

The GC modification procedure differs only in the first step, where the specific linker (poly-L-lysine) for carbon materials is used. Compared to gold modification, GC performance is less effective as redox peaks are visible until the BSA step. The most substantial peaks drop and redox potential differences are visible after polymer immobilization. The anti-RSV anchor results in a small return of the reversible behavior of [Fe(CN)_6_]^3−/4−^ that can be attributed to the lower net charge difference between the protein and ferro-/ferricyanide redox couple [33]. The following BSA adsorption results in a redox current decrease but no further ΔE change.

Electrochemical impedance spectroscopy (EIS) experiments are performed to evaluate the impact of consecutive steps of modifying the electrode on the impedance spectrum. EIS spectra of the bare and modified electrodes are recorded in 5 mM K_3_[Fe(CN)_6_]/K_4_[Fe(CN)_6_]/0.01 M PBS at the formal potential of the redox couple (0.150 V for Au and 0.211 V for GC). The measurements were conducted in a frequency range between 10 kHz and 1 Hz, and the spectra are presented in the form of a Nyquist plot. The results were simulated using the equivalent electrical circuit (Randles circuit) R_e_[CPE(R_ct_Z_W_)] **(**Figure 3B,D, inset) consisting of charge transfer resistance (R_ct_), electrolyte resistance (R_e_) combined in series with the constant phase element (CPE), and R_ct_. Diffusion in the low-frequency region is presented as the Warburg element (Z_W_). The semicircle diameter corresponds to the R_ct_ value, which is expected to increase at each step of surface modification. This parameter was used to compare modification levels for electrodes. Calculated data are presented in Table 1. For the Au modified surface, we observe the following increases in R_ct_. The greatest %R_ct_ change is for the anti-RSV step, which corresponds to the considerable size of antibodies and their anchor to the Au_4-ATP_GA surface (Figure 3B). In the case of GC, similarly to the CV data, one difference is seen in the GC_PLL and GC_PLL_anti-RSV stages, as the first impedance is higher than the second one (Figure 3D). Similarly to Au, the highest R_ct_ value was obtained for the BSA step. This value represents the resistance for a fully prepared biosensor right before the sample is added to the system. The gradual increase in resistance with each modification step indicates the electron transfer hindrance in the electrolyte biolayer phase due to its thickness increase. The CV and EIS results show agreement that the Au and GC electrodes’ surfaces have been successfully modified.

### 3.2. RSV Detection with Electrochemical Immunosensors

**Figure 4:**
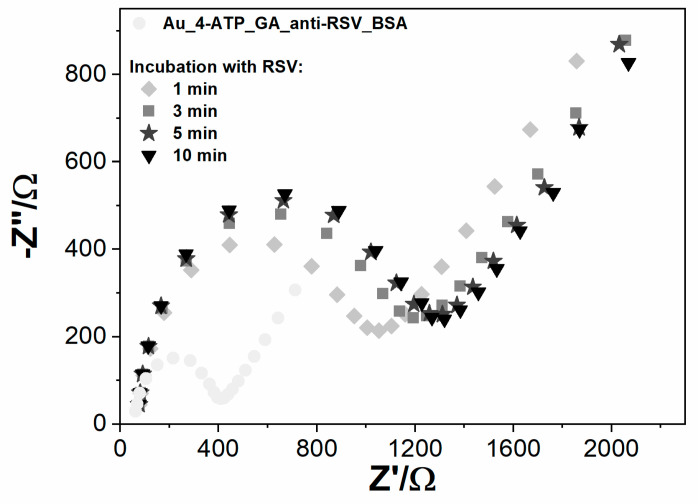
The impedance spectra registered for Au modified with anti‑RSV-bovine serum albumin (BSA), incubated for different periods (1–10 min) with RSV, registered in 5 mM K_3_[Fe(CN)_6_]/K_4_[Fe(CN)_6_]/0.01 M PBS.

Fully prepared electrochemical sensors were used for the detection of RSV. Due to differences in the Au and GC electrode design, the detection step was slightly different in both cases. On the Au/4-ATP/anti-RSV/BSA immunosensor, the sample of RSV, dissolved in 5 mM K_3_[Fe(CN)_6_]/K_4_[Fe(CN)_6_]/0.01 M PBS, was dropped onto the modified electrode surface, and impedance spectra were recorded in time (Figure 4). In the case of GC/PLL/anti-RSV/BSA, the RSV sample dissolved in 0.01 M PBS was firstly incubated on the electrode surface for a given time. Next, it was rinsed with PBS and immediately immersed in a fresh 5 mM K_3_[Fe(CN)_6_]/K_4_[Fe(CN)_6_]/0.01 M PBS solution for EIS measurement. Table 2 presents the GC-based immunosensor response after incubation in the RSV sample, expressed as charge transfer resistance change (ΔR_ct_). All R_ct_ change values were calculated from the formula:

$$\Delta R_{ct}=\frac{R_{ct_{S}}-R_{ct_{B}}}{R_{ct_{B}}}\times 100\%\;\left(2\right)$$

where $R_{ct_{S}}$ is the sample and $R_{ct_{B}}$ is a fully prepared immunosensor.

Developed sensing systems were checked in time cycles of 1, 3, 5, and 10 min incubations. The positive sample contains 6.3 × 10^6^ PFU/mL RSV. The observable change of R_ct_ was after 1 min of incubation on both immunosensor types. Optimal detection time was determined to be 5 min; longer incubation time did not significantly improve electrode response.

The Au electrode charge transfer resistance value increased 181.8% over the BSA measurement due to the 1 min incubation of RSV. The second measurement after 5 min of incubation resulted in an R_ct_ increase of 234.52%. The third spectrum after 10 min of incubation practically remained unchanged compared to the 5 min incubation (Figure 4). A similar tendency was observed for GC electrodes. With longer electrode incubation time with the RSV sample, the charge transfer resistance increased, and for 5 min, the sensing system reached 42.9% R_ct_ change, which is close to R_ct_ obtained for 10 min (Table 2). The results suggest that the binding of RSV to the antibodies creates a barrier to the electrode’s electrochemical process. After 5 min, the system was in equilibrium, which means that all antibodies were saturated with antigen. The 5 min incubation time was chosen for the detection time, and the relative R_ct_ parameter change was used as the primary response parameter.

The limit of detection (LOD) was determined using serial dilutions of the virus sample. LOD values were calculated from the relation LOD = 3 × SD/b, where SD is the standard deviation in the low concentration range, equal to 0.5 fg (*n* = 10), and b is the calibration curve slope. Linearity ranges and LODs of different methods for RSV detection are presented and compared in Table 3.

**Figure 5:**
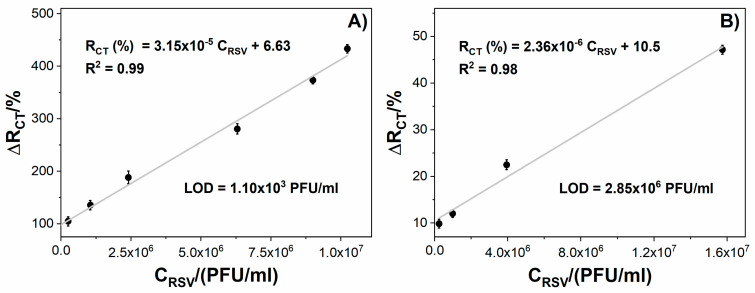
The relation between the immunosensor response expressed as R_ct_ change (ΔR_ct_) and the virus protein concentration for: (**A**) Au/4-ATP/anti-RSV/BSA, (**B**) GC/PLL/anti-RSV/BSA. Registered in 5 mM K_3_[Fe(CN)_6_]/K_4_[Fe(CN)_6_]/0.01 M PBS. Error bars denote confidence interval (α = 0.05, *n* = 3).

Figure 5 displays the impedance changes for tested immunosensors incubated with different RSV concentrations. The Au impedance immunosensor shows a linear relationship between the R_ct_ change and RSV concentration within the range of 1.0 × 10^5^ PFU/mL to 1.0 × 10^7^ PFU/mL with a correlation coefficient (R^2^) equal to 0.99 (Figure 5A). In the case of GC, the electrode shows a linear response from 1.0 × 10^5^ PFU/mL to 1.5 × 10^7^ PFU/mL with R^2^ = 0.98 (Figure 5B). The GC-based immunosensor show a wider linear dynamic range than the Au immunosensor; however, the LOD obtained for the modified Au was three orders of magnitude lower than the LOD for the GC electrode. A much lower LOD for the Au-based compared to GC-based sensor could have resulted from the lower intrinsic resistivity of the gold electrode and the influence of the crosslinker type used for surface coverage. The sensitivity is 3.15 × 10^−5^ %(PFU/mL)^−1^ for Au and 2.36 × 10^−6^ %(PFU/mL)^−1^ for GC (Figure 5).

**Figure 6:**
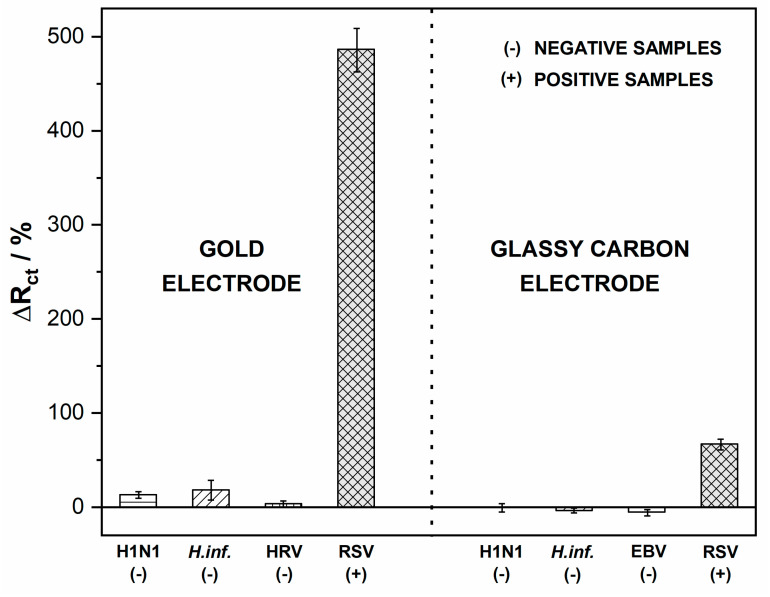
Impedance spectra recorded after incubation with RSV and interfering pathogens. Registered in 5 mM K_3_[Fe(CN)_6_]/K_4_[Fe(CN)_6_]/0.01 M PBS, the incubation time was 5 min. Error bars denote confidence interval (α = 0.05, *n* = 3).

After verifying the signal alteration after the RSV binding to the electrode surface, the platform’s usability was tested on the pathogen samples. To validate the biosensors’ response in a real environment, we simulated throat conditions by incubating the biosensing electrodes with various common throat-infecting pathogens, including Haemophilus influenzae, rhinovirus (HRV), Epstein–Barr virus (EBV), and H1N1 influenza A virus. They were used as potentially interfering pathogens to investigate the selectivity of the immunosensors. The samples were incubated with the tested immunosensors for 5 min, and unbound viral particles were flushed away with deionized water. Next, the EIS spectra were recorded and relative ΔR_ct_ values were calculated. Direct coupling of RSV antigen caused an impedance increase in both the Au biosensor and GC biosensor. The percentage change of R_ct_ for negative samples did not exceed 15% (Figure 6). For negative samples, the charge transfer resistance did not change significantly, which proves no unwanted interaction of the bacteria with the biosensing electrode surface. The small decrease in R_ct_ values in the cases of Haemophilus influenzae and EBV samples on GC could have been due to a high load of non-specific bacteria. The above results confirmed the specificity of both developed sensors in the presence of negative buffers and positive RSV samples. The negative samples were proved to give no cross-reactivity, indicating that the proposed methods have high selectivity for detecting RSV (Figure S2).

## 4. Conclusions

In this work, we describe the development of two different impedimetric sensing platforms to detect RSV. One of the developed immunosensors is based on a gold electrode, coated with an antibody attached through a 4-ATP crosslinker. The second immunosensor utilizes the GC electrode surface for antibody binding and poly-L-lysine as a crosslinker. We compare the performance of immunosensors in terms of their sensitivity, limit of detection, dynamic range, and specificity for RSV. The viral detection is based on the analysis of electrochemical impedance spectroscopy spectra recorded at the electrodes’ antibody-modified surfaces. The LOD of 1.1 × 10^3^ PFU/mL makes the Au immunosensor competitive with other RSV detection methods. The LOD obtained for GC is three orders of magnitude higher than the LOD for Au, however, the GC immunosensor shows a dynamic range of 1.0 × 10^5^ PFU/mL to 1.5 × 10^7^ PFU/mL, more than for Au (1.0 × 10^5^ PFU/mL to 1.0 × 10^7^ PFU/mL). The lower LOD and higher sensitivity of the Au immunosensor are probably due to a higher density of anti-RSV antibody coverage on the electrode surface, which strongly depends on the modification method. Developed immunoassays did not cross-react with other pathogens, including influenza A virus (H1N1), human rhinovirus, Epstein–Barr virus, and Haemophilus influenzae bacteria. The gold electrode revealed excellent electrochemical properties and ease of surface modification; thus, it can be a fast, facile, and highly selective testing platform. To summarize, the proposed Au-based impedimetric biosensor shows better analytical performance in terms of sensitivity and LOD for RSV than the GC-based biosensor.

## Figures and Tables

**Table 1:** List of values of elements calculated from the equivalent electric circuit (EEQC) for bare and modified electrodes.

**Gold Electrode**				
**Modification Step**	**Re (Ω)**	**R_ct_ (Ω)**	CPE ^1*^ (µΩ^−1^s^n^)	***n***
Au	15.58	129.8	0.33	0.980
Au_4-ATP	42.15	221.3	1.60	0.980
Au_4-ATP_GA	57.72	328.4	9.63	0.944
Au_4-ATP_GA_anti-RSV	59.92	612.5	6.12	0.951
Au_4-ATP_GA_anti-RSV_BSA	54.38	784.1	9.33	0.947
**Glassy Carbon Electrode**				
**Modification Step**	**Re (Ω)**	**R_ct_ (Ω)**	**CPE (µΩ^−1^s^n^)**	***n***
GC	72.19	40.06	45.9	0.687
GC_PLL ^2*^	78.19	1457	15.6	0.874
GC_PLL_anti-RSV	72.59	925.8	14.3	0.862
GC_PLL_anti-RSV_BSA	76.46	1708	23.1	0.883

^1*^ Constant phase element; ^2*^ poly-L-lysine.

**Table 2:** Values of R_ct_ change obtained for modified GC electrodes for different time periods (1–10 min) registered in 5 mM K_3_[Fe(CN)_6_]/K_4_[Fe(CN)_6_]/0.01 M PBS. ΔR_ct_ values were averaged from three repetitions.

Incubation Time	ΔR_ct_/%
1 min	12.2 (±1.66)
3 min	20.6 (±1.00)
5 min	42.9 (±2.38)
10 min	43.7 (±1.83)

**Table 3:** A comparison of the analytical characteristics of the immunosensors developed in this work with relevant immunosensors for RSV detection based on the literature.

Type of Method	Detection Limit	Year	Reference
UV–Vis spectroscopy	2.11 × 10^2^ PFU/mL	2016	[34]
RT-PCR	1.79 × 10^1^ PFU/mL	2013	[35]
ELISA	5.0 × 10^1^ PFU/mL	1982	[36]
RT-qPCR	1.0 × 10^1^ PFU/mL	2003	[18]
Surface Enhanced Raman Spectroscopy(SERS)	1.00 × 10^2^ PFU/mL	2006	[37]
Potentiometry (immunosensor)	10^3^ PFU/mL	2013	[38]
Fluorimetry	1.19 × 10^1^ PFU/mL	2009	[39]
Electrochemical impedance spectroscopy (Au/4-ATP/anti-RSV/BSA)	1.10 × 10^3^ PFU/mL	2020	This work
Electrochemical impedance spectroscopy (GC/PLL/anti-RSV/BSA)	2.85 × 10^6^ PFU/mL	2020	This work

## Supplementary Materials

The following are available online at https://www.mdpi.com/2079-6374/10/11/175/s1, Figure S1: The standard curve of RSV quantification from qPCR, Figure S2: Impedance spectra recorded after incubation with RSV and interfering pathogens. Registered in 5 mM K3[Fe(CN)6]/K4[Fe(CN)6]/0.01 M PBS..
