# Supplementary material for: Electrochemical Immunosensors Based on Screen-Printed Gold and Glassy Carbon Electrodes: Comparison of Performance for Respiratory Syncytial Virus Detection
Source: Biosensors (Basel). 2020 Nov 13;10(11):175. doi: 10.3390/bios10110175 (PMC7698328; doi:10.3390/bios10110175)
Supplement: Supplementary file 1 [file biosensors-10-00175-s001.pdf]

Supplementary Material

Electrochemical immunosensors based on screen-printed gold and glassy carbon electrodes. Comparison of performance for Respiratory Syncytial Virus detection.

Wioleta Białobrzeska [1,2,*], Daniel Firganek [1], Maciej Czerkies [3], Tomasz Lipniacki [3], Marta Skwarecka [1], Karolina Dziąbowska [1,2], Zofia Cebula [1], Natalia Malinowska [2], Daniel Bigus [1], Ewelina Bięga [1], Krzysztof Pyrć [4], Katarzyna Pala [2], Sabina Żołędowska [1,2], Dawid Nidzworski [1,2]

[1] Institute of Biotechnology and Molecular Medicine, 3 Trzy Lipy St., 80-172 Gdansk, Poland
[2] SensDx, 14b Postępu St., 02-676 Warszawa, Poland
[3] Institute of Fundamental Technological Research Polish Academy of Sciences, Pawińskiego 5B, 02-106 Warszawa, Poland
[4] Malopolska Centre of Biotechnology, Jagiellonian University, Gronostajowa 7, 30-387 Krakow, Poland
* Correspondence: e-mail: wioletta.bialobrzeska@etongroup.eu

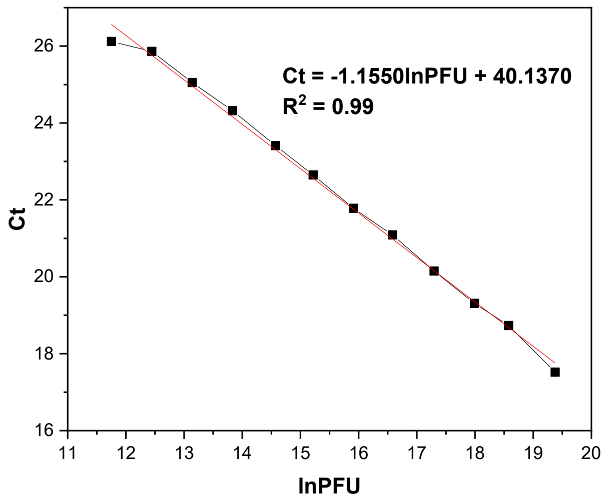

Figure S1. The standard curve of RSV quantification from qPCR.

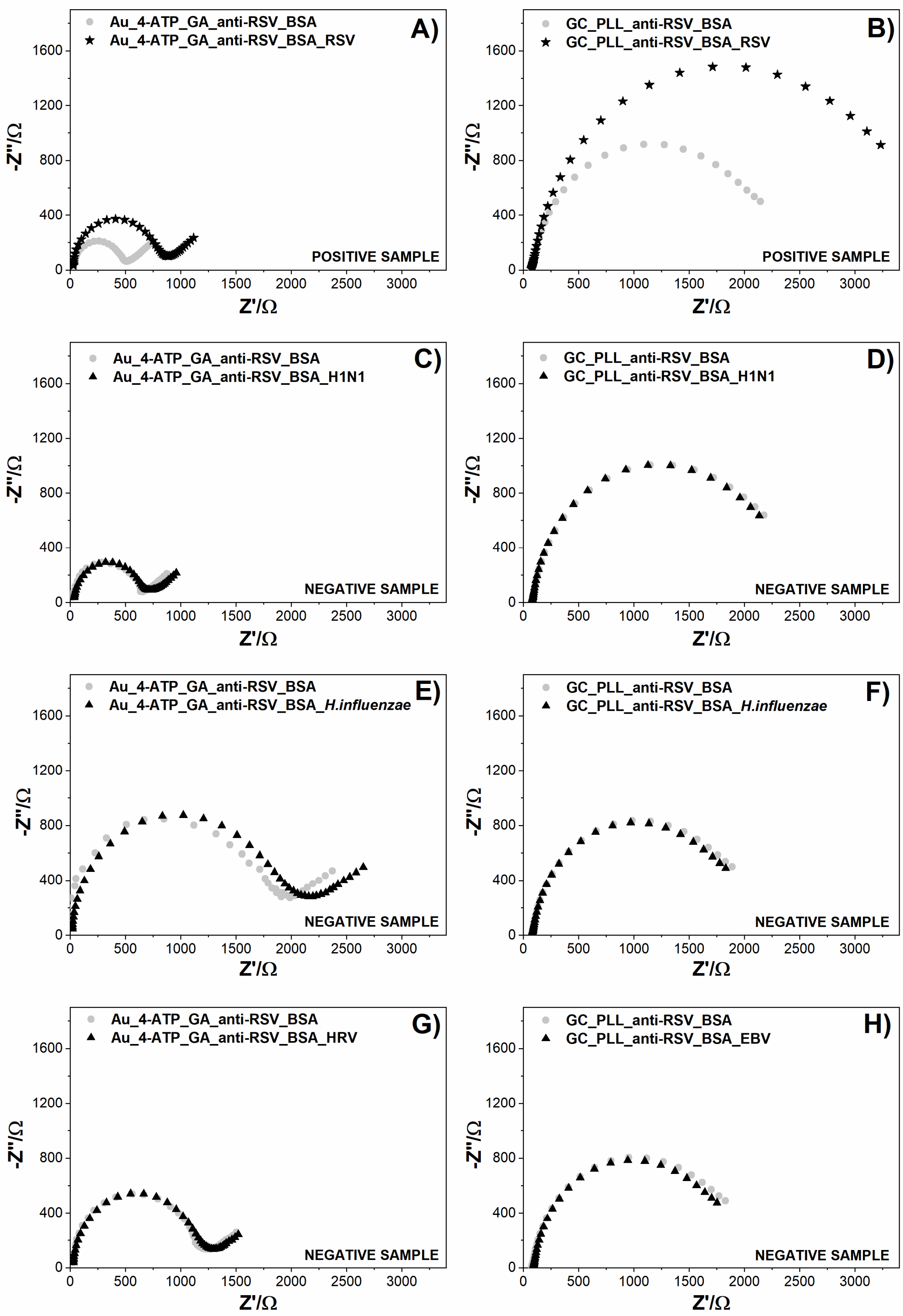

Figure S2. Impedance spectra recorded after incubation with RSV and interfering pathogens. Registered in 5 mM $K_3[Fe(CN)_6]/K_4[Fe(CN)_6]$/0.01 M PBS.
